# Supplementary material for: An Angiogenesis-Related lncRNA Signature Is Associated with Prognosis and Tumor Immune Microenvironment in Breast Cancer
Source: J Pers Med. 2023 Mar 13;13(3):513. doi: 10.3390/jpm13030513 (PMC10057494; doi:10.3390/jpm13030513)
Supplement: Supplementary file 1 [file jpm-13-00513-s001.zip › jpm-2254338-supplementary figures.pdf]

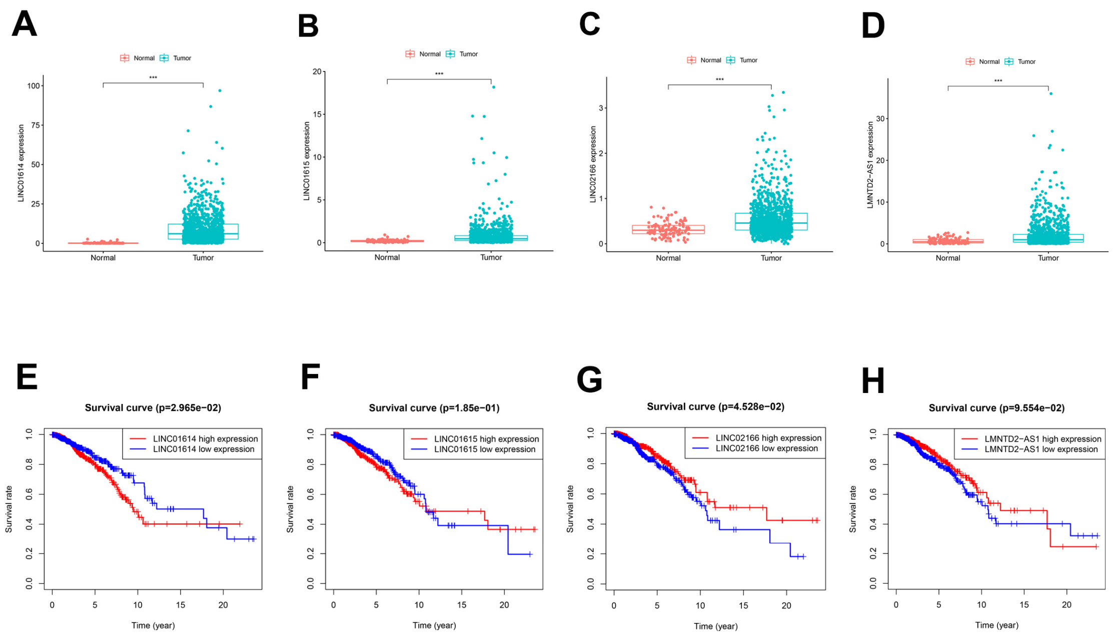

Figure S1. Expression of AR-lncRNAs in ARlncM in breast cancer tissue and its influence on the prognosis of breast cancer patients (A-H).

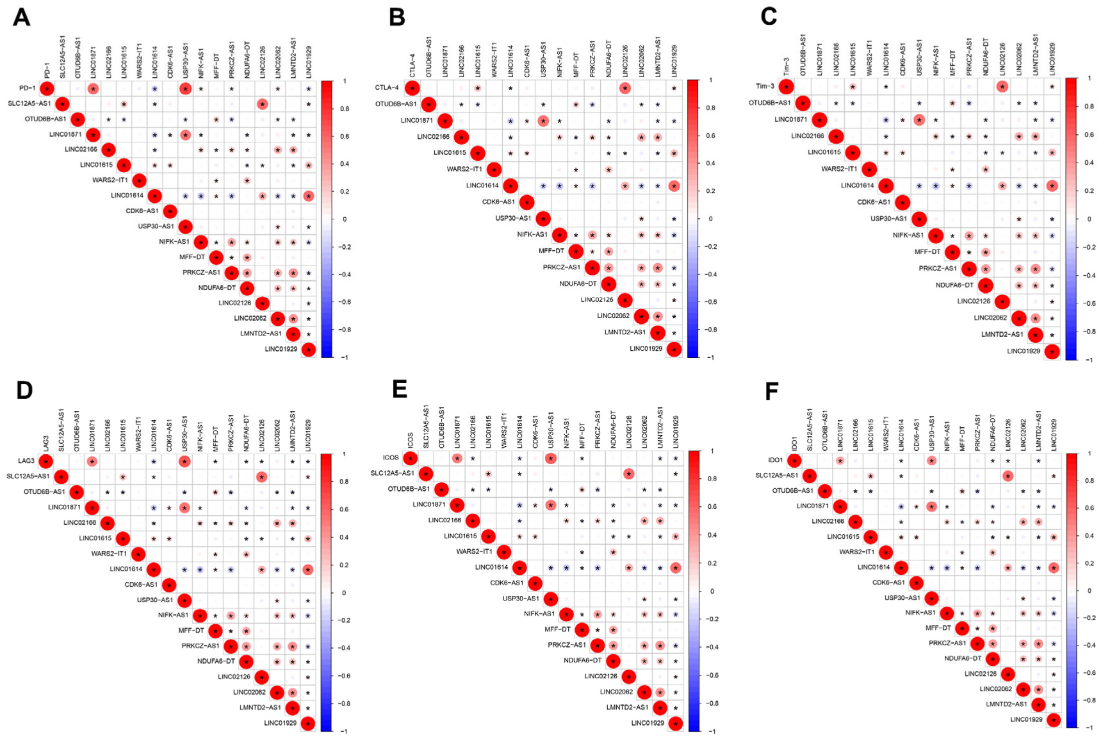

Figure S2. Correlation of 17 prognostic-related AR-lncRNAs with PD-1 (A), CTLA-4 (B), Tim-3 (C), LAG3 (D), ICOS (E), and IDO1 (F).
